# Complete genome sequencing of Peyer’s patches-derived *Lactobacillus taiwanensis* CLG01, a potential probiotic with antibacterial and immunomodulatory activity

**DOI:** 10.1186/s12866-021-02127-z

**Published:** 2021-02-27

**Authors:** Xiao-yu Li, Li-xiang Li, Yan Li, Ru-chen Zhou, Bing Li, Xiang Gu, Shi-chen Fu, Bi-ying Jin, Xiu-li Zuo, Yan-qing Li

**Affiliations:** 1 Department of Gastroenterology, Qilu Hospital, Cheeloo College of Medicine, Shandong University, 107 Wenhuaxi Road, Jinan, 250012 Shandong Province China; 2 Laboratory of Translational Gastroenterology, Qilu Hospital, Cheeloo College of Medicine, Shandong University, Jinan, Shandong China; 3 Robot engineering laboratory for precise diagnosis and therapy of GI tumor, Qilu Hospital, Cheeloo College of Medicine, Shandong University, Jinan, Shandong China

**Keywords:** *Lactobacillus taiwanensis*, Probiotic, Antibacterial activity, Immunomodulation, Peyer’s patches, complete genome

## Abstract

**Background:**

The genus *Lactobacillus* is an important component of the gastrointestinal tract of human and animals and commonly considered as probiotic. *L. taiwanensis* has long been proposed to be a probiotic whereas understanding on this species is still in its infancy. Genomic information of *L. taiwanensis* is fairly limited. Extensive characterization of its beneficial traits is needed.

**Results:**

A new strain CLG01 of *L. taiwanensis* was isolated from mouse Peyer’s patches. We established its probiotic profile through in vitro experiments. Complete genome of this strain was also sequenced and analyzed. *L. taiwanensis* CLG01 showed robust tolerance to acid and a degree of tolerance to bile salt with a promising antibacterial activity against a broad spectrum of pathogenic bacteria. In vitro treatment of mouse RAW 264.7 macrophage cells with heat-killed bacteria and bacterial supernatant of *L. taiwanensis* CLG01 resulted in enhancement of immune responses and upregulated expression of TNF-α and IL-6. The strain CLG01 also increased the IL-10 production of macrophages when co-treated with lipopolysaccharide (LPS). Complete genome of *L. taiwanensis* CLG01 contained a 1.89 Mb chromosome and two plasmids. Further genomic analysis revealed the presence of genes related to its resistance to different stresses and the beneficial effects mentioned above. Moreover, biosynthetic gene clusters (BGCs) encoding antimicrobial peptides, like bacteriocin, linear azol(in)e-containing peptide (LAP) and lanthipeptide, were also identified in the genome of *L. taiwanensis* CLG01.

**Conclusions:**

*L. taiwanensis* CLG01, isolated from mouse Peyer’s patches, is the first *L. taiwanensis* strain with both phenotypes and genotypes systematically studied. These preliminary data confirmed the role of *L. taiwanensis* CLG01 as a potential probiotic candidate with antibacterial and immunomodulatory activity, which provide insight for further investigation to this species.

**Supplementary Information:**

The online version contains supplementary material available at 10.1186/s12866-021-02127-z.

## Background

Mammalian gastrointestinal tract is a nutrient-rich environment populated by diverse, highly mutualistic microbes [1]. Gut microbes and their bioactive byproducts have profound impacts on health status and gut homeostasis of the host [2]. Accumulating evidence demonstrated that abnormal host-microbial interactions could be the central or contributing cause of multiple enteral and parenteral diseases, including inflammatory bowel disease (Crohn’s disease and ulcerative colitis) [3], colorectal carcinoma [4–6], neurodegenerative disorders (e.g., Parkinson’s disease and Alzheimer’s disease) [7, 8], metabolism disorders (e.g., obesity and type 2 diabetes) [9]. Therefore, it’s imperative to maintain the balance of the interplay between gut microbiota and the host. In recent years, microbial-based strategies (e.g., probiotics, prebiotics, and fecal microbiota transplants) aiming at modulating gut microbiomes have been tested in a wide range of clinical contexts, with satisfying outcomes obtained [10, 11].

Probiotics are defined as “live microorganisms that, when administered in adequate amounts, confer a health benefit on the host” according to the World Health Organization (WHO) [12]. Lactic acid bacteria (LAB) are a group of Gram-positive bacteria that produce lactic acid as a major fermentation metabolite [13]. The genus *Lactobacillus* is the most studied LAB and specific *Lactobacillus* species are regarded as probiotic due to a wide range of benefits. *Lactobacilli* exerts beneficial effects on intestinal epithelia by direct contact with epithelial cells [14]. *L. fermentum* was reported to initiate signalling pathways involved in epithelial barrier protection in the mouse model of colitis [15]. Furthermore, previous studies have shown that some heat-killed *Lactobacillus* strains (e.g., *L. rhamnosus* GG, *L. helveticus* IMAU70129, and *L. casei* IMAU60214) could trigger innate immune responses and improve the phagocytic and bactericidal activities of human macrophages [16]. In addition, certain strains of *L. helveticus* and *L. acidophilus* were capable of reducing the growth of HT*-*29 human colon cancer cells and subsequently exert an anti-tumor effect [17]. Some types of *Lactobacillus* species proved to be probiotics*,* such as *L. acidophilus, L. rhamnosus* GG, have been commercially utilized in dairy products and bring therapeutic benefits to the host [18, 19]. Of note, the probiotic properties can be species-, dose-, and disease-specific and may not be the same as other strains of the same genus or species [20]. Consequently, searches for new probiotics and further exploitation of the existing ones are still necessary.

*L. taiwanensis* was originally isolated and identified by Taiwanese from silage cattle feed and showed the highest sequence homology with *L. gasseri* and *L. johnsonii* [21, 22]. Previous studies have shown that *L. taiwanensis* is abundant in small intestine [23]. The BALB/c mice treated with *L. taiwanensis* display an elevated Treg cell frequency in the gut-associated lymphoid tissues [24]. Researches based on the 16S rRNA sequencing have revealed the low abundance of *L. taiwanensis* in colorectal cancer and cervical neoplasia, indicating that *L. taiwanensis* might provide protective effects in the colorectal tumorgenesis and cervical neoplasia development [25, 26]. Kim et al. have reported that *L. taiwanensis* is capable of producing antimicrobial peptides and inhibiting growth of pathogenic microorganisms like *Salmonella gallinarum* and *Streptococcus iniae* [27]. These scattered examples indicate that *L. taiwanensis* possibly represent a novel probiotic species. However, few data are available to support this view. *L. taiwanensis* has not yet been functionally characterized. Despite the significance in predicting the structures and important capabilities of microorganisms, the current genome information of *L. taiwanensis* is fairly limited, with only one strain genetically accessible. In this study, a new strain of *L. taiwanensis*, named *L. taiwanensis* CLG01, was isolated from the Peyer’s patches, the lymphoid tissues of mouse small intestine. We evaluated some basic physiological characteristics, including the ability to survive in acid and bile salt, adhesive ability, antibacterial activity, and immunomodulatory effects in vitro. By detailed analysis of the complete genome sequence of *L. taiwanensis* CLG01, we are allowed to combine the beneficial effects of CLG01 with its genomic features.

## Results

### *Lactobacillus* isolation and identification

A total of 9 strains were isolated with the de Man Rogosa Sharpe (MRS) agar plates from mouse Peyer’s patches samples. All isolates were Gram-positive with rod shape under optical microscopy. On the basis of 16S rRNA sequencing and analysis, the isolates were identified as *L. taiwanensis* (*n* = 1), *L. reteuri* (*n* = 4), *L. johnsonii* (*n* = 3) *and L. murinus* (*n* = 1) respectively*.* The *L. taiwanensis* strain, named as CLG01, was selected for further investigations of physiological functions and probiotic traits. Single colony of *L. taiwanensis* CLG01 was distinguishable as translucent with irregular border after 48 h of incubation on MRS agar plate (Fig. S1). Scanning electron microscopy (SEM) image of this strain revealed a typical rod-like structure (Fig. S2).

### Genome features and phylogenetic analysis of *L. taiwanensis* CLG01

**Fig. 1:**
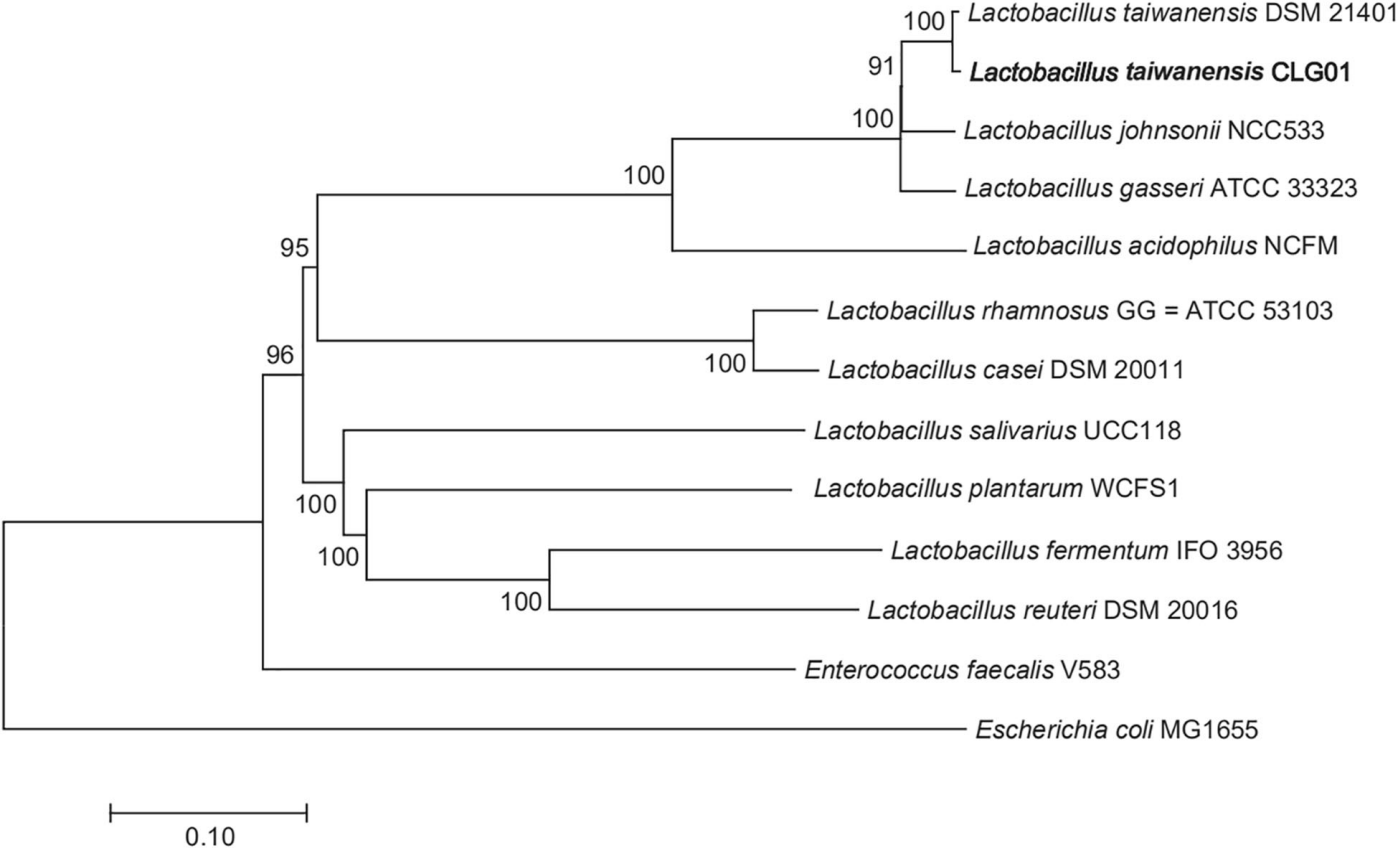
Phylogenetic tree of *L. taiwanensis* CLG01. The tree was constructed using neighbor-joining method based on the whole genome sequences. Genome sequence data were obtained from the NCBI. The strain *Enterococcus faecalis* V580 and *Escherichia coli* MG1655 were used as out groups. Bootstrap values at nodes are percentages of 1000 replicates

**Fig. 2:**
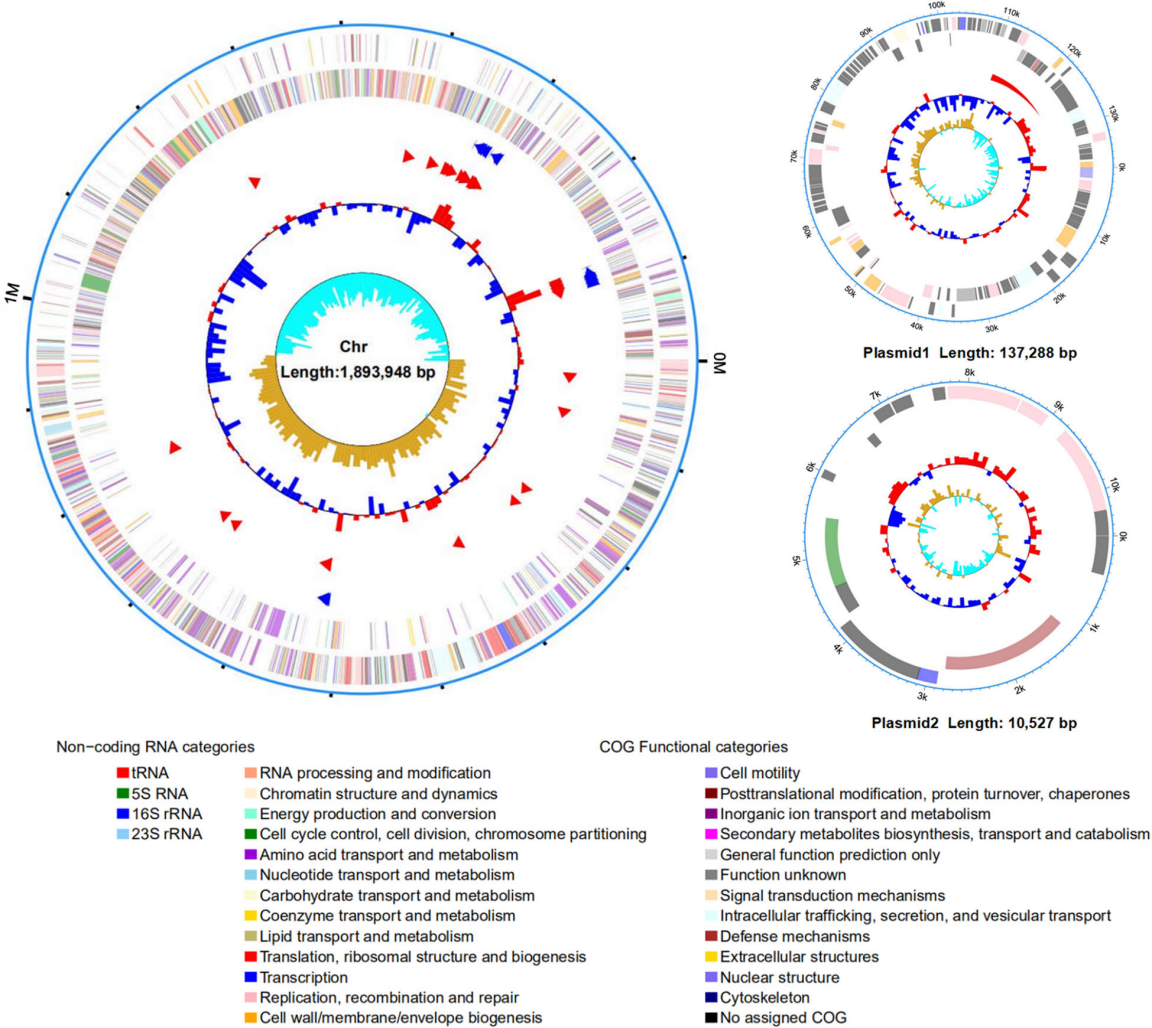
Circular genome maps of *L. taiwanensis* CLG01 chromosome and the plasmids. From outside to the center: 1. forward CDSs colored by COG functional categories; 2. reverse CDSs colored by COG function categories; 3. rRNA; 4. tRNA; 6. GC content; and 7. GC skew. The circular plots were drawn with R package circlize

**Fig. 3:**
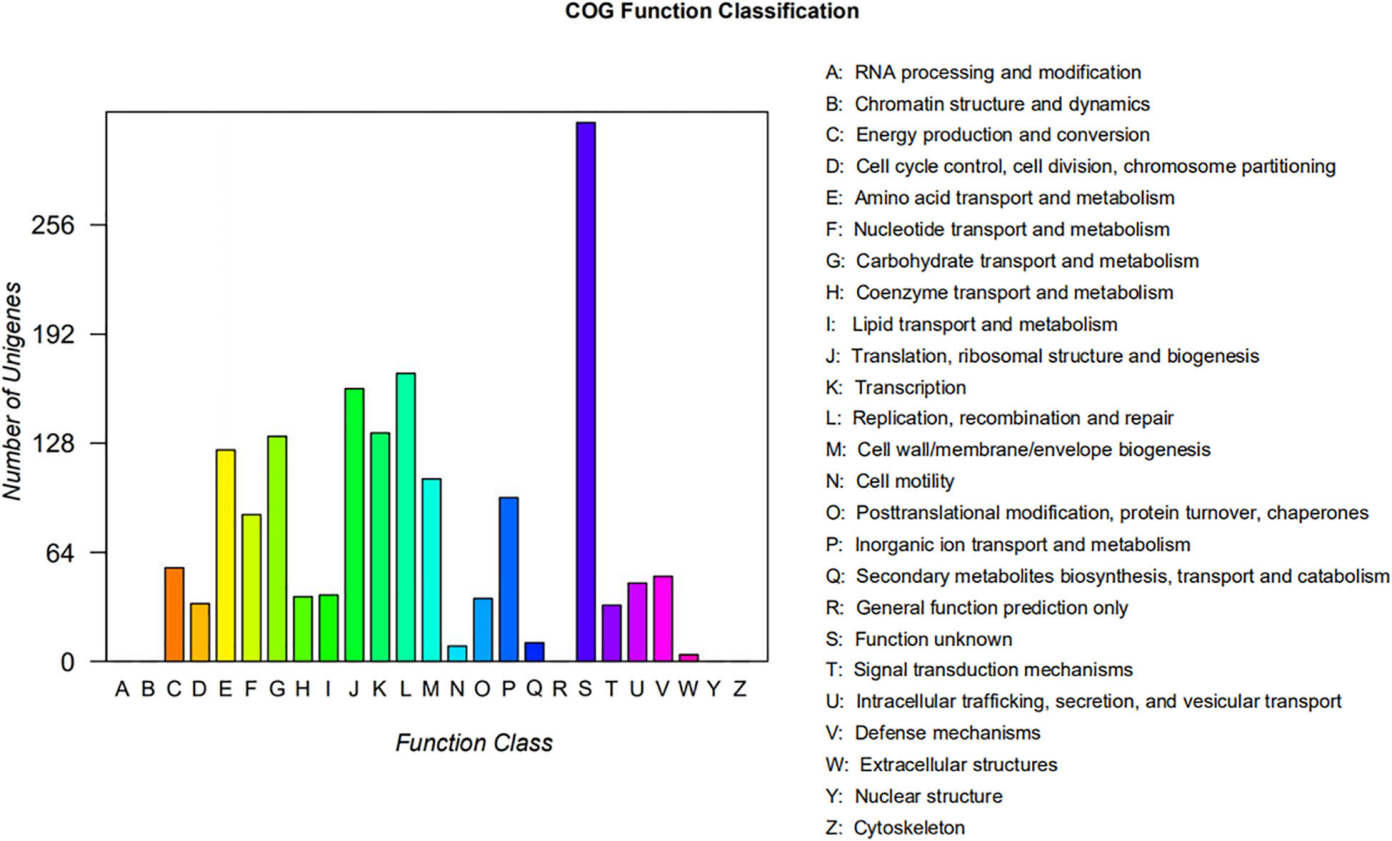
Distribution of genes across COG functional categories in the genome of *L. taiwanensis* CLG01

To further elucidate the genomic characteristics of *L. taiwanensis* CLG01, complete genome sequencing was performed. The whole genome of *L. taiwanensis* CLG01 was composed of a 1.89 Mb single, circular chromosome with GC content of 34.11% and two circular plasmids named as plasmid1 (137,288 bp) and plasmid2 (10,527 bp). A total of 1994 genes (1771 protein-coding genes), 21 rRNA genes, and 77 tRNA genes were identified (Table 1). To unravel the phylogenetic position of the strain CLG01, a phylogenetic tree based on 295 single-copy orthologous genes was constructed using neighbor-joining method (Fig. 1). The genome-wide phylogenetic analysis of CLG01 showed the closest relatedness with *L. taiwanensis* DSM 21401. Circular genome map of *L. taiwanensis* CLG01 revealed the genome distribution (Fig. 2). After searching for clusters of orthologous groups (COG) database, 1680 protein-coding genes were classified functionally into 25 categories (Fig. 3, Table S1). “Function unknown” has the highest number of genes, followed by “Replication, recombination, and repair,” “Translation, ribosomal structure and biogenesis” and “Transcription”. Additionally, through the analysis of the whole genome by the webtool PathogenFinder, the *L. taiwanensis* CLG01 was predicted to be a non-human pathogenic microorganism.

**Table 1:** General genome features of *L. taiwanensis* CLG01

Attributes	Values
Genome size (bp)	1,893,948
GC content (%)	34.11%
Plasmid	2
rRNAs	
5S rRNA	7
16S rRNA	7
23S rRNA	7
tRNAs	77
ncRNAs	3
Protein-coding genes	1771
Pseudo genes	122

### Carbohydrate metabolism of *L. taiwanensis* CLG01

Among the COG-recognized genes, 132 genes involved in carbohydrate metabolism and transport with 55 genes for energy production and conversion. Moreover, 19 genes encoding proteins of phosphotransferase system (PTS), a major carbohydrate active transport system, were detected within the genome of CLG01 (Table S2). PTS in CLG01 was involved in transporting various types of sugars, including glucose, beta-glucoside, galactitol, cellobiose, lactose, mannose, fructose, sorbose, glucitol, and sorbitol. A total of 73 genes predicted carbohydrate-active enzymes (CAZymes) were identified in the *L. taiwanensis* CLG01 genome and divided in to 5 subgroups including 30 genes for glycosyltransferases, 24 genes for glycoside hydrolases, 8 genes for carbohydrate esterases, 8 genes for carbohydrate-binding molecules and 3 genes for auxiliary activity.

### Stress resistance analysis

**Fig. 4:**
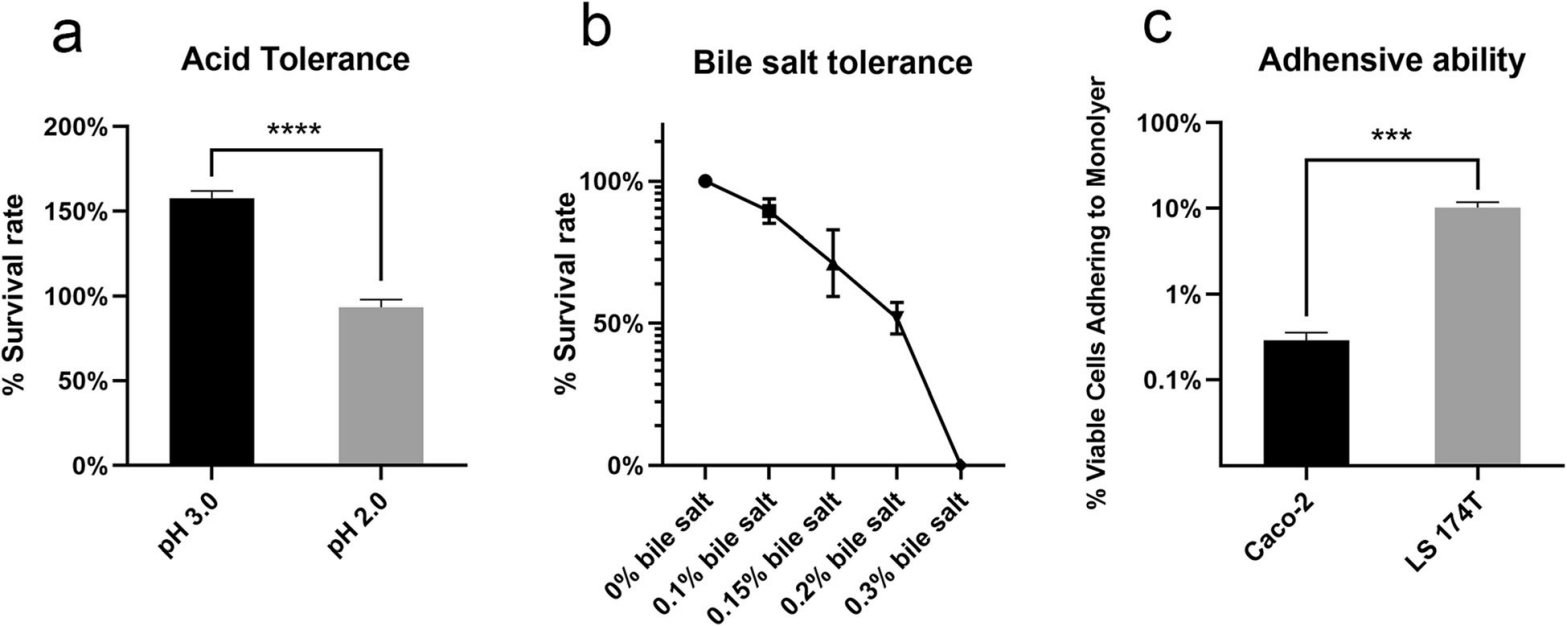
Stress resistance and adhesive ability of *L. taiwanensis* CLG01. **a** Survival rate of CLG01 in the condition of pH 3.0 and pH 2.0.**b** Survival rate of CLG01 in the medium containing different concentrations of ox gall sodium cholate. **c** The percentage of viable cells adhering to Caco-2 and LS 174 T monolayers. Data expressed as mean ± SD from three repeated experiments. Relevant statistically significant differences are as follow: ∗∗∗*P* < 0.001 and ∗∗∗∗*P* < 0.0001

In the aim of characterizing the potential of *L. taiwanensis* CLG01 to survive in the harsh environment of gastrointestinal tract, we studied its ability to tolerant acid and bile salt in vitro. Viable cells were measured after incubation for 2 h in low pH conditions. Considerable acid tolerance was observed at both pH 3.0 and pH 2.0 (Fig. 4a). In detail, GLG01 could stay alive and increase beyond the initial count at pH 3.0 and maintain a high survival rate of 93.3 ± 4.63% at pH 2.0. Results of bile salt tolerance were shown in Fig. 4b. The strain CLG01 exhibited a survival rate of 89.4 ± 4.26% on the medium containing 0.1% bile salt. With the increase of ox gall sodium cholate concentration, the growth of CLG01 was gradually impaired. However, no obvious colony was observed on the MRS agar plates with the bile salt concentration of 0.3%, suggesting that the bacterial growth was totally inhibited.

At molecular level, genome of CLG01 was predicted to carry the gene encoding sodium-proton antiporter which exchange sodium ions and protons across the membrane [28] and 8 genes encoding F0F1-ATP synthase which is essential to maintain intracellular pH homeostasis [29] (Table 2). The CLG01 harbored the gene (H1A07_00295) that encode linear amide C-N hydrolase, which belongs to choloylglycine hydrolase family and catalyzes the de-conjugation of bile acids [30]. More stress-related proteins linked to the resistance to different types of stresses (e.g., temperature, oxidative stress) were also identified in the genome of *L. taiwanensis* CLG01 with detailed information listed in Table 2.

**Table 2:** Stress-related proteins of *L. taiwanensis* CLG01

Stress factors	Stress-related proteins	Locus tag
Temperature	Molecular chaperone DnaJ	H1A07_03770
	Molecular chaperone DnaK	H1A07_03765
	Molecular chaperone HtpX	H1A07_00400
	Molecular chaperone HslO	H1A07_01505
	Chaperonin GroEL	H1A07_02245
	Co-chaperone GroES	H1A07_02240
pH	Sodium-proton antiporter	H1A07_00580
	F0F1-ATP synthase	H1A07_05945, H1A07_05950, H1A07_05955, H1A07_05960, H1A07_05970, H1A07_05975, H1A07_05975, H1A07_05980
Bile salt	Linear amide C-N hydrolase	H1A07_00295
Oxidative stress	Lactate oxidase	H1A07_09050
	Thiol peroxidase	H1A07_04795
	Thioredoxin	H1A07_02340
	Pyruvate oxidase	H1A07_09225

### Adhesive ability of *L. taiwanensis* CLG01

Bacterial adhesive ability to host intestinal epithelium and the outer mucus layer is one of the selecting criteria for probiotics [31]. We tested the host attachment capability of *L. taiwanensis* CLG01 in vitro. Two kinds of human intestinal epithelial cells Caco-2 and LS 174 T monolayers were used. As shown in Fig. 4c, *L. taiwanensis* CLG01 adhered much better to LS 174 T cells than Caco-2 cells with two orders of magnitude difference observed (*P* < 0.001). The bioinformatic analysis results showed that the CLG01 genome encoded the cell-surface proteins that involved in adhesion such as CBS domain-containing protein (H1A07_01450 and H1A07_08900), elongation factor Ts (H1A07_03665), and elongation factor Tu (H1A07_05600), etc.

### Antibacterial activity of *L. taiwanensis* CLG01

**Fig. 5:**
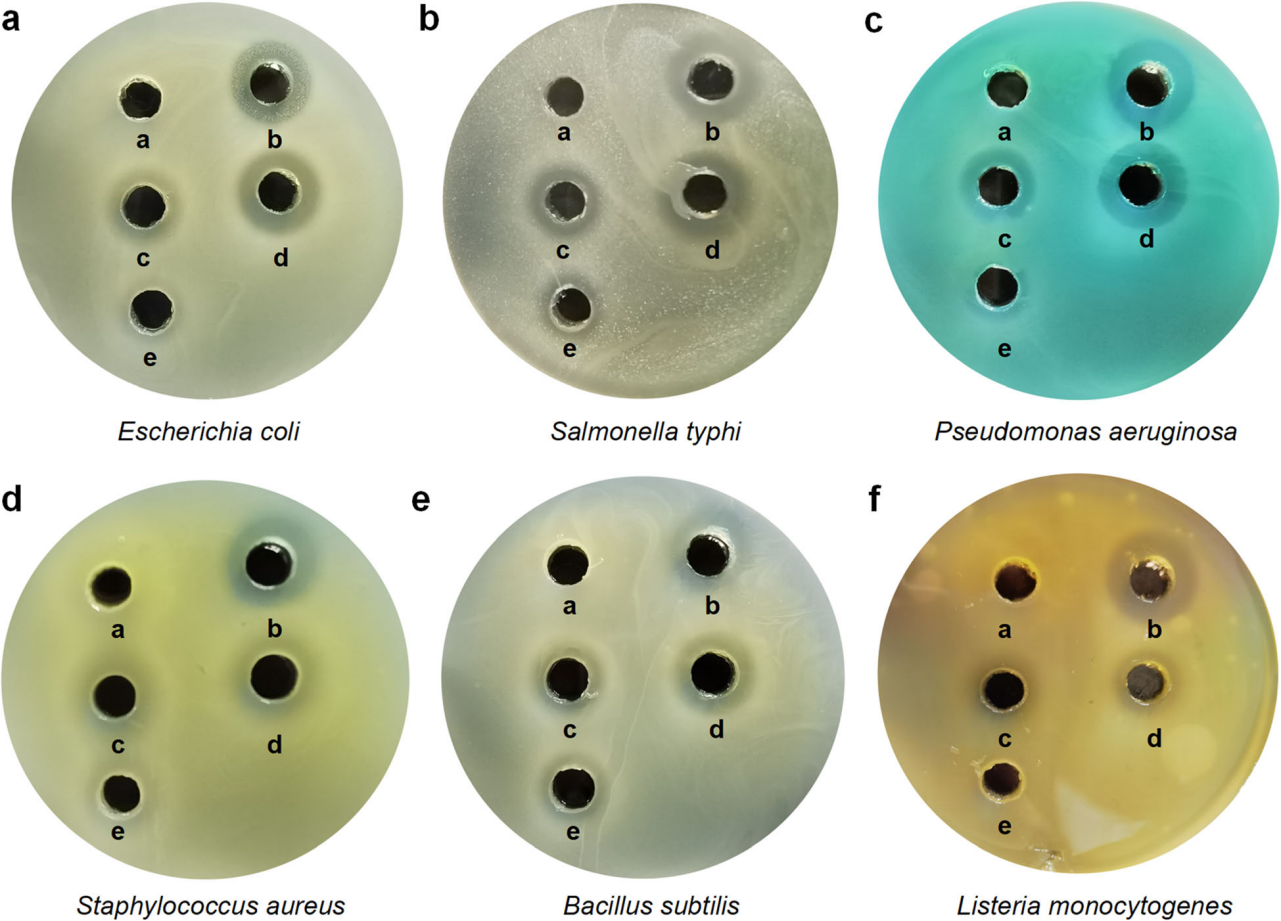
Inhibitory effect of *L. taiwanensis* CLG01 on the growth of pathogenic microorganisms. Inhibition zones were obtained from the following treatments: **a**, MRS broth; **b**, Ampicillin or gentamycin; **c**, Cell-free supernatant of *L. taiwanensis* CLG01; **d**, Cell-free supernatant of *L. taiwanensis* CLG01 pretreated with proteinase inhibitor EDTA; **e**, Cell-free supernatant of *L. taiwanensis* CLG01 pretreated with proteinase

One of the important characteristics of probiotics is to antagonize pathogens. In this study, the antibacterial activity of *L. taiwanensis* CLG01 was evaluated by well agar diffusion method. The cell-free supernatant of *L. taiwanensis* CLG01 was obtained at different time points (12 h,16 h and 20 h). The diameters of inhibition zones were measured after 24 h incubation at 37 °C and the results are reported in Table 3. Our results showed that the cell-free supernatant of *L. taiwanensis* CLG01 exhibited inhibitory effects against both Gram-positive bacteria (*Staphylococcus aureus, Listeria monocytogenes and Bacillus subtilis*) and Gram-negative bacteria (*Escherichia coli, Salmonella typhi and Pseudomonas aeruginosa*) with a digestive tropism. The strain CLG01 showed the strongest antibacterial activity (> 15 mm) against *Staphylococcus aureus*, while the weakest result was for *Listeria monocytogenes*. The cell-free supernatant obtained at 16 h has the strongest inhibitory effect on the growth of indicator bacteria. To further determine the type of the inhibitory substances responsible for the anti-pathogen activity, the cell-free supernatant was pretreated by proteinase or proteinase inhibitor EDTA respectively. We found that proteinase pretreatment led to significant decreases of inhibition zones around the wells, whereas EDTA treatment enlarged the circle diameters (Fig. 5).

**Table 3:** Antibacterial activity of *L. taiwanensis* CLG01 against pathogenic bacteria

Indicator bacteria	Inhibition zone (mm)		
	12 h Supernatant	16 h Supernatant	20 h Supernatant
*Escherichia coli*	12.16 ± 0.74	12.60 ± 0.68	11.71 ± 0.43
*Salmonella typhi*	10.93 ± 0.85	12.09 ± 0.21	11.30 ± 0.23
*Pseudomonas aeruginosa*	13.52 ± 0.47	14.72 ± 0.28	12.90 ± 0.46
*Staphylococcus aureus*	15.21 ± 0.57	15.82 ± 0.62	13.30 ± 1.01
*Bacillus subtilis*	12.36 ± 0.42	13.53 ± 0.81	13.20 ± 0.74
*Listeria monocytogenes*	8.81 ± 0.21	11.08 ± 0.16	9.89 ± 0.62

Inhibition zone diameters are expressed as mean ± SD in mm obtained from three independent experiments

**Fig. 6:**
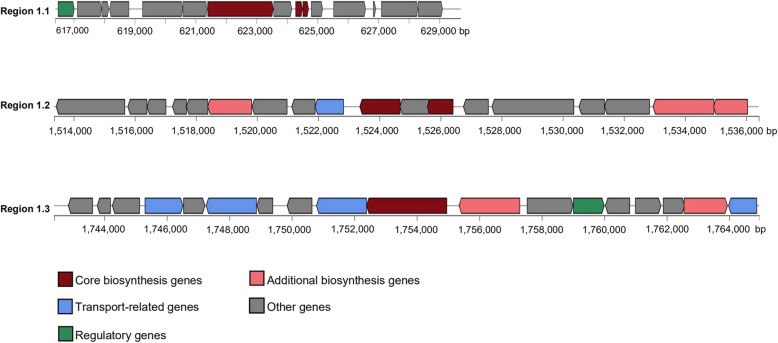
Predicted biosynthetic gene clusters encoding secondary metabolites in the *L. taiwanensis* genome. The gene clusters encoding bacteriocin (Region 1.1), LAP (Region 1.2), and lanthipeptide (Region 1.3) are represented by arrows with different colors corresponding to the operons of different functions.

Consistent with the observed inhibitory effects of *L. taiwanensis* CLG01, the genome of CLG01 contained genes coding for antibacterial compounds, like bacteriocins (H1A07_02945 and H1A07_02950). Bacteriocin immunity proteins (H1A07_02990 and H1A07_03010) were identified in the genome as the self-protecting factors. Furthermore, analysis of secondary metabolites BGCs using the antiSMASH database revealed the presence of BGCs related to bacteriocin, LAP and lanthipeptide biosynthesis (Fig. 6). The BGC encoding bacteriocin was composed of 15 genes and exhibited a high sequence similarity of 77% with gassericin T, a broad-spectral bacteriocin produced by *L. gasseri* [32]*.* Detailed gene information of the three BGCs were shown in Table S3, S4, S5.

### Immunomodulatory effects of *L. taiwanensis* CLG01 in murine macrophage model

**Fig. 7:**
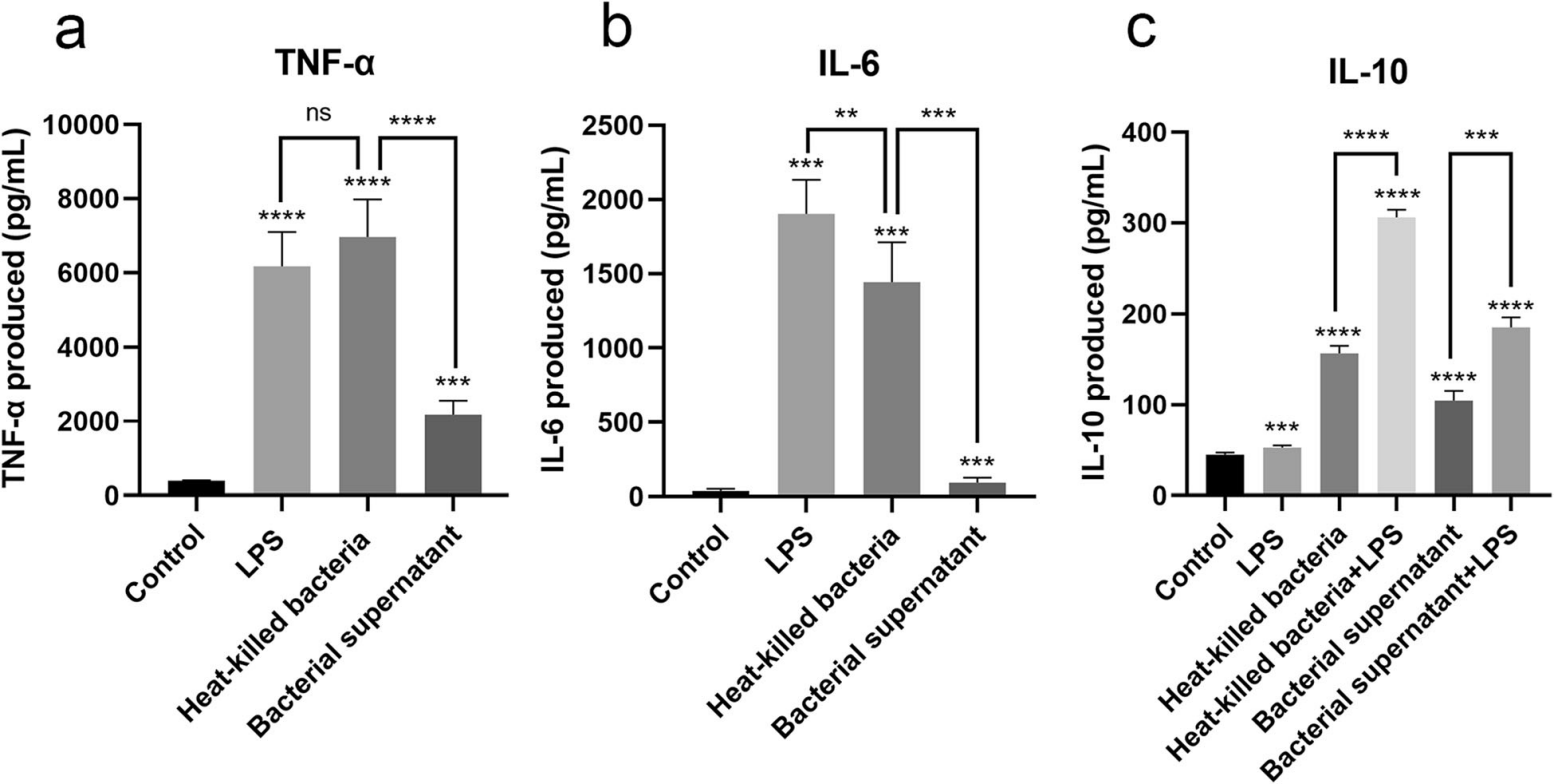
Induction of cytokines by *L.taiwanensis* CLG01 in RAW 264.7 macrophage cells. TNF-α(**a**) and IL-6 (**b**) induced by heat-killed bacteria and bacterial supernatant of *L. taiwanensis* CLG01 in RAW 264.7 macrophages. **c** Induction of IL-10 by heat-killed bacteria and bacterial supernatant of *L. taiwanensis* CLG01 in RAW 264.7 macrophages upon LPS challenge. Data expressed as mean ± SD from three repeated experiments. Statistical significance in the figures are as follows: * *P* < 0.05, ∗∗*P* < 0.01, ∗∗∗*P* < 0.001, and ∗∗∗∗*P* < 0.0001; ns, no significant difference

To quantitatively assess the response of macrophages to *L. taiwanensis* CLG01, the production of cytokines TNF-α and IL-6 from macrophages was examined by enzyme-linked immunosorbent assay (ELISA). The RAW 264.7 cells released high amounts of both cytokines upon the treatment of LPS. Compared to the control, both heat-killed bacteria and bacterial supernatant of *L. taiwanensis* CLG01 markedly increased the production of TNF-α (Fig. 7a) and IL-6 (Fig. 7b) of RAW 264.7 macrophages after 12 h of incubation. For heat-killed bacteria treatment, TNF-α level appeared to be higher than those of LPS-treated group. However, no statistical significance was observed. IL-6 level was significantly lower than those of LPS-treated group (*P* = 0.0033). In addition, the effect of bacterial supernatant on production of both cytokines was much weaker than heat-killed bacteria.

In order to evaluate the anti-inflammatory function of CLG01, production of anti-inflammatory cytokine IL-10 was measured in standard and LPS-treated conditions (Fig. 7c). Co-treatment with LPS was used to simulate the inflammatory condition. The results showed that both heat-killed bacteria (*P* < 0.0001) and bacterial supernatant (*P* < 0.0001) of *L. taiwanensis* CLG01 markedly increased the IL-10 production in the absence of LPS. IL-10 level of both groups was shown to be greater than LPS-only treated group. Importantly, statistically significant increase in the IL-10 production was observed when 1 μg/mL LPS co-treatment was administered (*P* < 0.001).

Further genomic analysis showed that *L. taiwanensis* CLG01 encoded proteins that involved in exopolysaccharide (EPS), peptidoglycan (PG), and polysaccharide biosynthesis, including exopolysaccharide biosynthesis protein (H1A07_05520, H1A07_05530) and peptidoglycan endopeptidase (H1A07_09465). Additionally, CLG01 encode two putative LTA synthase family proteins (H1A07_06055, H1A07_07705) that involved in the biosynthesis of lipoteichoic acid (LTA).

## Discussion

Genomic analysis combined with experimental studies provide an approach to study the characteristics of probiotics comprehensively. *L. taiwanensis* has been postulated to be a probiotic by many researchers [25–27], whereas supportive evidence is scarce. In the present study, a new strain was isolated from Peyer’s patches of healthy mouse small intestine and identified as *L. taiwanensis* CLG01 based on 16S rRNA sequencing. The strain CLG01 was characterized for probiotic features, including the acid and bile salt tolerance, adhesion ability, antibacterial activity and in vitro immunomodulatory effects. The *L. taiwanensis* CLG01 displayed a degree of resistance to acid and bile salt with in vitro antibacterial activity and could enhance the innate immune responses in RAW 264.7 macrophages cells. Complete genome sequencing and analysis of *L. taiwanensis* CLG01 provided molecular biology clues for these beneficial effects.

*Lactobacillus* species of intestinal origin are considered intrinsically resistant to acidic environment. However, the acid tolerant properties vary widely among species [33]. *L. taiwanensis* CLG01 showed robust survival in low pH environments. The survival rates of CLG01 in pH 2.0 and pH 3.0 are consistent with some well-studied probiotic strains, like *L. johnsonii* ATCC 33200 and *L. johnsonii* 456. The strain CLG01 survived better than the probiotic strain *L. acidophilus* ATCC 4356 in pH 2.0 [34]. The presence of stress-related proteins in the genome and the high ability to metabolize carbohydrate may facilitate its resistance. Given the protective effects of metabolizable sugars on *Lactobacilli* in acidic environment, *L. taiwanensis* CLG01 may survive better in the fermented food [35]. *L. taiwanensis* was detected as one of the most abundant *Lactobacillus* species in the butter [36].

Although CLG01 could remain viable at bile salt concentration between 0 and 0.2%, it was sensitive to 0.3% ox gall sodium cholate. The strain CLG01 only showed moderate bile salt tolerance relative to other probiotic strain, like *L. plantarum* 299 V [34]. This is probably due to its specific niche environment. Morphologically, the Peyer’s patches in mouse small intestine showed a typical dome-shaped structure, which was defined as the subepithelial dome [37]. Compared to the small intestinal lumen, the concentration of bile salt is much lower at this site, from which the strain CLG01 was derived.

Similar to other reported probiotic *Lactobacillus* species, the adhesive ability of *L. taiwanensis* CLG01 to Caco-2 was lower than that to LS 174 T [34], which may be due to their distinct mucin profiles. Caco-2 cells express transmembrane mucins such as MUC1 and MUC3, whereas LS 174 T cells express a great amount of MUC2, which is the major secreted mucin that comprise intestinal mucus layer and provide a common binding site for commensal bacteria [38]. Furthermore, the in vivo intestinal environment is more complex than in vitro with syntrophic interactions between members of microbial communities. Lin et al. have reported the commensalism between *L. reuteri* and *L. taiwanensis,* in which *L. reuteri* enhances the colonization ability of *L. taiwanensis* in mouse gastrointestinal tract through coaggregation [39].

Probiotics control pathogenic infections through producing antimicrobial peptides, generating organic acid to lower local pH, or enhancing functions of innate immune system [40–42]. In line with the finding reported by Kim et al. [27], the cell-free supernatant of *L. taiwanensis* CLG01 was also observed to limit the growth of both Gram-positive and Gram-negative pathogens to different degrees and the antimicrobial effect could be inhibited by the treatment of proteinase, indicating that the inhibitory effects might arise from proteinaceous substances. The in silico screen resulted in the identification of gene clusters encoding bacteriocin, LAP and lanthipeptide, which could at least partially explain its antimicrobial activity. Antibiotic resistance has become a global crisis in recent years and pose a threat to public health. New antibiotics alternatives are in urgent need. Bacteriocins produced by food-grade LAB are of great interests due to its heat stability and the inhibitory effects against pathogenic bacteria, which may facilitate their application in food preservation and treatment of infection [43]. The acidocin B derived from *L. acidophilus* and the helveticin J from *L. helveticus* are remarkable examples of bacteriocins produced by *Lactobacillus* strains [44]. The pediocin produced by *Pediococcus* strains has been approved by the FDA [45]. Lanthipeptides are a class of ribosomally synthesized and posttranslationally modified peptides (RiPPs) with characteristic lanthionine structures and show promising antimicrobial action against various Gram-positive pathogens and selective Gram-negative pathogens [46]. Certain lantibiotics (e.g., nisin and haloduracin) have been used together with traditional antibiotics to fight against multidrug-resistant bacteria [47]. Functional genome mining can offer an effective approach to identify new members of this family and their biosynthetic machinery. In this sense, *L. taiwanensis* may serve as a new source of bioactive antibacterial compounds. Further work will be done to confirm the expression of these genome-predicted metabolites and establish their biosynthesis pathway as well as detailed mode of action.

We intended to screen the *Lactobacillus* strains with immunomodulating capacity in this study. Peyer’s patches, the gut-associated lymphoid follicles, are inductive sites for mucosal immune responses [48] and may serve as reservoir for unique microorganisms with immunomodulatory property. It has been reported that specific *L. reuteri* colonized within mouse Peyer’s patches could increase the cytokines production of RAW 264.7 cells [49] and modulate the secretion of secretory immunoglobulin A [50].

Macrophages are major participants in innate immunity and the most rapid cell type to respond to pathogens’ invasion [51]. TNF-α and IL-6 are important cytokines involved in activation of macrophages and recruitment of immune cells [52, 53]. In this work, heat-killed bacteria and the supernatant containing soluble metabolites of *L. taiwanensis* CLG01 were capable of activating RAW 264.7 macrophage cells. The effect of heat-killed bacteria was more marked, indicating that the immune responses mostly result from surface cell components of CLG01. It has been established that extracellular proteins of *Lactobacillus* species can elicit immune responses through activating distinct pattern recognition receptors present in epithelial cells and immune cells. LTA is the major microbe-associated molecular pattern of Gram-positive bacteria and the agonist of Toll-like receptor 2 [54], while PGs are more likely to activate NOD-like receptors [55]. The presence of genes related to LTA and PG biosynthesis provide a clue to the mechanism of macrophages activation. In particular, *L. taiwanensis* CLG01 could induce the secretion of IL-10 in vitro, a potent anti-inflammatory cytokine essential for restraining excessive inflammatory and immune responses [56]. The combined treatments of strain CLG01 with LPS resulted in a greater IL-10 secretion, suggesting that *L. taiwanensis* CLG01 might exert a greater anti-inflammatory effect in the context of inflammation. Previous studies have reported the anti-inflammatory activity of EPS and the role it played in macrophages activation [57, 58]. Although in vitro models cannot fully represent the real situation, the results obtained from co-culture assays can be predictive of the in vivo immunomodulatory properties of *lactobacilli*. For further therapeutic application, animal models with diverse immunological status should be established to determine the effectiveness of the Peyer’s patches-derived *L. taiwanensis* CLG01*.*

## Conclusion

In the current study, we systematically characterized the probiotic properties of *L. taiwanensis* for the first time and disclosed its complete genome sequence. The strain CLG01 of *L. taiwanensis*, isolated from Peyer’s patches, showed the ability to survive in acid and bile salt. Moreover, CLG01 exhibited inhibitory effects against multiple pathogens and induced the immune responses of macrophages in vitro. This study will provide a considerable insight into the physiological functions of *L. taiwanensis* and the corresponding molecular mechanisms and consequently contribute to its application in producing novel antibiotics and regulating intestinal immunity.

## Methods

### Isolation and culture of the bacteria strain

Peyer’s patches samples were collected from small intestine of healthy C57BL/6 mice. The study was approved by the Ethical Committee on Scientific Research of Shandong University Qilu Hospital (DWLL-2020-003). All animal experiments have followed ARRIVE guidelines. We confirm that all methods were carried out in accordance with relevant guidelines and regulations. The Peyer’s patches samples were fully homogenized and ten-fold serial dilutions of the homogenates were spread onto MRS agar plates (Qingdao Haibo Biotechnology Company, Qingdao, China) and incubated at 37 °C for 48 h under microaerophilic condition. Single colonies were selected based on their colony morphologies and purified by re-streaking on MRS agar plates. The isolates were Gram-stained. SEM (Zeiss, Oberkochen, Germany) was used to observe the bacterial morphology. Bacterial genomic DNA was extracted and the V3, V4 region of 16S rRNA gene was amplified through PCR using primers 27F (5′-AGAGTTTGATCMTGGCTCAG-3′) and 1492R (5′-TACGGYTACCTTGTTACGACTT-3′) as described before [59]. The amplification products were visualized using 1% agarose gel electrophoresis and submitted for sequencing. The sequences obtained were compared to those in National Center Biotechnology Information (NCBI) database using nucleotide BLAST tool. Isolates were stored in MRS broth containing 20% (v/v) glycerol at − 80 °C.

### Complete genome sequencing and assembly and annotation

Bacterial cell pellet was harvested by centrifugation and total DNA was extracted using E.Z.N.A® Bacteria DNA kit (Omega Bio-tek, Norcross, USA). Complete genome was sequenced with a combination of Illumina platform (Illumina, TX, USA) and Oxford Nanopore platform (Oxford Nanopore Technologies, Oxford, UK). The reads were assembled using ABySS 2.0 [60]. GeneMarkS [61] was adopted for the prediction of protein-coding RNA in the whole genome. The prediction of rRNA and tRNA were proceeded by RNAmmer 1.2 [62] and tRNAscan-SE 2.0.4 [63]. Functional gene annotation was based on NCBI non-redundant database [64], COG database [65], and CAZymes database [66]. Genome sequences of *L. taiwanensis* CLG01 have been submitted to GenBank under accession number CP059276 (chromosome), CP059277 (plasmid1), and CP059278 (plasmid2).

### Phylogenetic tree and bioinformatic analysis

Ten strains of *Lactobacillus* species, one strain of *Enterococcus faecalis* and one strain of *Escherichia coli* with complete genomes were downloaded from NCBI database. Single copy, orthologous genes shared by all species were selected and multiple-sequence alignments of these genes were performed using MAFFT [67]. Aligned sequences were concatenated to obtain whole-genome-wide alignment for phylogenetic analyses. A neighbor-joining tree was generated and rooted by FastTree [68]. Bootstrap values were obtained by running 1000 bootstrap replicates. Secondary metabolites gene clusters were predicted using antiSMASH 5 [69]. The PathogenFinder (https://cge.cbs.dtu.dk//services/ResFinder/) was used to estimate the pathogenicity of CLG01.

### Acid and bile salt tolerance assays

Acid tolerance assay was performed as described before [70] with little modification.

MRS broth was adjusted to pH 2.0 and 3.0 with 1 M hydrochloric acid. *L. taiwanensis* CLG01 was cultured overnight in MRS broth at 37 °C and bacterial suspension (1 × 10^8^ cells/mL) was inoculated into acidified MRS broth and cultured for 2 h. After incubation, bacterial suspension was serial diluted, plated on MRS agar and incubated at 37 °C for 48 h. Survival rates (%) was calculated by comparing final count of viable cells with the initial count. To test the bile salt tolerance of *L. taiwanensis* CLG01, bacterial suspension was serially diluted and plated onto MRS agar plates containing 0–0.3% (w/v) ox gall sodium cholate (Sangon Biotech, Shanghai, China). Inoculated agar plates were incubated at 37 °C for 48 h and the viable cells were counted. Bile salt tolerance was determined by calculating the ratio (%) of viable cells compared control without bile salt [71].

### Adhesion assay

The adhesion assay was performed as described before [34]. Human colorectal adenocarcinoma cell line Caco-2 and human goblet cell line LS 174 T were purchased from American Type Culture Collection (ATCC) and grown in Dulbeccos modified Eagles medium (DMEM) containing 10% fetal bovine serum (FBS) and 1% penicillin-streptomycin at 37 °C in a 5% CO_2_ atmosphere. Caco-2 cells and LS 174 T cells were seeded respectively in 12-well plates at 5 × 10^5^ cells/well and grown to confluent monolayers. Overnight cultures of *L. taiwanensis* CLG01 were centrifugated, rinsed, and suspended in antibiotic-free DMEM to a concentration of 2–5 × 10^8^ cells/mL. Cell monolayers were washed twice with sterile phosphate buffered saline (PBS). A 1 mL of suspended bacterial sample was added to each well. After 2 h of incubation, supernatants were removed and the monolayers were washed with PBS for 3 times to remove the non-adherent bacteria. Then cell monolayers were agitated vigorously with micropipette tips and suspended in 1 mL PBS. The resulting suspensions were serially diluted and plated on MRS agar plates. The agar plates were incubated at 37 °C for 48 h and the percentage of viable cells were calculated.

### Antibacterial activity test

The antibacterial activity of *L. taiwanensis* CLG01 was determined by well agar diffusion method described by Zheng et al. [72] with little modification. Standard strains of pathogenic bacteria were purchased from China Medical Microorganism Culture Collection (CMCC), including *Escherichia coli* CMCC 44102, *Staphylococcus aureus* CMCC 26003, *Pseudomonas aeruginosa* CMCC 10104, *Bacillus subtilis* ATCC 6633, *Salmonella typhi* CMCC 50071, and *Listeria monocytogenes* ATCC 19115. These strains were used as the indicator bacteria and cultured in Luria-Bertani (LB) broth overnight at 37 °C with shaking at 200 rpm. *L. taiwanensis* CLG01 were cultured in MRS broth at 37 °C, with aliquots taken at 12, 16, and 20 h of incubation. The cell-free supernatant of CLG01 was obtained by centrifugating at 8000 g for 15 min and filtering via 0.22-μm filters. LB agar was autoclaved and cooled to a temperature of 45 °C. Then 10% (v/v) indicator bacteria liquid was added. Approximately 25 mL LB agar was poured over a 9.0 cm petri dish and allowed to solidify at room temperature. Wells were punched on the plates with 200 μL cell-free supernatant added into each well. An equal volume of fresh MRS broth was used as a negative control. Ampicillin (20 μg/mL) served as the positive control for *Escherichia coli*, *Staphylococcus aureus*, *Salmonella typhi* and *Listeria monocytogenes*, while gentamycin (20 μg/mL) served as the positive control for *Pseudomonas aeruginosa* and *Bacillus subtilis*. After incubation at 37 °C for 24 h, diameters of inhibition zones were measured. Additionally,1 mg/mL pepsin enzyme and 5 mM EDTA were used to pretreat the cell-free supernatant for 2 h respectively. The mixtures were assayed for antibacterial activity as described above.

### Cytokine induction in mouse RAW 264.7 macrophages cells

Bacterial cell pellet of *L. taiwanensis* CLG01 was harvested and washed, and the concentration was adjusted to 1 × 10^8^ cells/mL with sterile saline. Heat-killed bacteria was prepared by incubation in a metal bath at 100 °C for 15 min and resuspended in DMEM. For the preparation of bacterial supernatant, the bacteria were resuspended in DMEM and incubated at 37 °C for 2 h. Subsequently, bacteria were removed by centrifugation and filtration via a 0.22-μm filter. The supernatants were retained for next step experiment. Mouse macrophage cell line RAW 264.7 was purchased from ATCC and the culture condition was the same as Caco-2 cells and LS 174 T cells. Approximately 1 × 10^6^ cells were seeded in each well of 12-well cell culture plates and stabilized overnight. Heat-killed bacteria and bacterial supernatant were administered. Cells treated with 1 μg/mL LPS (Sigma-Aldrich, MO, USA) were used as positive control. Non-treated cells were used as negative control. After 12 h of treatment, supernatant of each group was collected and analyzed for TNF-α and IL-6 levels using ELISA (Lianke Biotech, Hangzhou, China) according to the manufacturers’ instructions.

### Anti-inflammatory effects of *L. taiwanensis* CLG01 upon LPS challenge

To determine the anti-inflammatory effects of CLG01, cells and bacteria preparations were the same as described above. Macrophage cells were treated with heat-killed bacteria and bacterial supernatant for 12 h in the presence or absence of 1 μg/mL LPS. Similarly, cells treated with 1 μg/mL LPS were used as positive control with the non-treated cells as negative control. IL-10 levels of the resulting supernatants were determined by ELISA.

### Statistical analysis

All experiments were repeated three times. Statistical analysis was performed by Graphpad Prism 8. Data was presented as the mean ± standard deviation (SD). Two-tailed, unpaired Student’s t tests were used to assess the significance of differences in acid tolerance and adhesion assays. Results of cytokines production were analyzed using non-parametric tests (Mann-Whitney test, Kruskal-Wallis test).

## Supplementary Information

**Additional file 1: Table S1.** COG function classification of encoding proteins in the genome of *L. taiwanensis* CLG01. **Table S2.** The phosphotransferase system (PTS) in the genome of *L. taiwanensis* CLG01. **Table S3.** Detailed information of the bacteriocin biosynthetic gene cluster in *L. taiwanensis* CLG01 genome. **Table S4.** Detailed information of the LAP biosynthetic gene cluster in *L. taiwanensis* CLG01 genome. **Table S5.** Detailed information of the Lanthipeptide biosynthetic gene cluster in *L. taiwanensis* CLG01 genome. **Figure S1.** Colony morphology of *L. taiwanensis* CLG01 on MRS agar plate after 48 h of incubation. **Figure S2.** Morphology of *L. taiwanensis* CLG01 under a scanning electron microscope (SEM).

## Data Availability

The dataset of complete genome sequencing of *L. taiwanensis* CLG01 have been deposited to National Center Biotechnology Information repository with accession number CP059276-CP059278.

## Abbreviations

LPS: Lipopolysaccharide
BGCs: Biosynthetic gene clusters
LAP: Linear azol(in)e-containing peptide
LAB: Lactic acid bacteria
MRS: De Man Rogosa Sharpe
SEM: Scanning electron microscopy
COG: Clusters of orthologous groups
PTS: Phosphotransferase system
CAZymes: Carbohydrate-active enzymes
ELISA: Enzyme-linked immunosorbent assay
EPS: Exopolysaccharide
PG: Peptidoglycan
LTA: Lipoteichoic acid
RiPPs: Ribosomally synthesized and posttranslationally modified peptides
NCBI: National Center Biotechnology Information
ATCC: American Type Culture Collection
DMEM: Dulbeccos modified Eagles medium
CMCC: China Medical Microorganism Culture Collection
FBS: Fetal bovine serum
PBS: Phosphate buffered saline
LB: Luria-Bertani
SD: Standard deviation
