# Supplementary material for: Complete genome sequencing of Peyer’s patches-derived Lactobacillus taiwanensis CLG01, a potential probiotic with antibacterial and immunomodulatory activity
Source: BMC Microbiol. 2021 Feb 27;21:68. doi: 10.1186/s12866-021-02127-z (PMC7916312; doi:10.1186/s12866-021-02127-z)
Supplement: Supplementary file 1 — Additional file 1: Table S1. COG function classification of encoding proteins in the genome of L. taiwanensis CLG01. Table S2. The phosphotransferase system (PTS) in the genome of L. taiwanensis CLG01. Table S3. Detailed information of the bacteriocin biosynthetic gene cluster in L. taiwanensis CLG01 genome. Table S4. Detailed information of the LAP biosynthetic gene cluster in L. taiwanensis CLG01 genome. Table S5. Detailed information of the Lanthipeptide biosynthetic gene cluster in L. taiwanensis CLG01 genome. Figure S1. Colony morphology of L. taiwanensis CLG01 on MRS agar plate after 48 h of incubation. Figure S2. Morphology of L. taiwanensis CLG01 under a scanning electron microscope (SEM). [file 12866_2021_2127_MOESM1_ESM.docx]

**Supplementary material for****:**

**Complete genome sequencing of *Lactobacillus taiwanensis* CLG01, a potential probiotic with antibacterial and immunomodulatory activity**

Xiao-yu Li^1,2^, Li-xiang Li^1,2^, Yan Li^1,2^, Ru-chen Zhou^1,2^, Bing Li^1,2^, Xiang Gu^1,2^, Shi-chen Fu^1,2^, Bi-ying Jin^1,2^, Xiu-li Zuo^1,2,3^, Yan-qing Li^1,2,3^

1.Department of Gastroenterology, Qilu Hospital, Cheloo College of Medicine, Shandong University，Jinan, Shandong, China

2.Laboratory of Translational Gastroenterology, Qilu Hospital, Cheloo College of Medicine, Shandong University, Jinan, Shandong, China

3. Robot engineering laboratory for precise diagnosis and therapy of GI tumor，Qilu Hospital, Cheloo College of Medicine, Shandong University, Jinan, Shandong, China

**Corresponding Author:** Dr. Yan-qing Li, Department of Gastroenterology, Qilu Hospital, Shandong University, 107 Wenhuaxi Road, Jinan 250012, Shandong Province, China; liyanqing@sdu.edu.cn; + 86 185 6008 7666

**Table S1 COG function classification of encoding proteins in the genome of *L. taiwanensis* CLG01.**

| **Category** | **COG description** | **Number of genes** |
| --- | --- | --- |
| A | RNA processing and modification | 0 |
| B | Chromatin structure and dynamics | 0 |
| C | Energy production and conversion | 55 |
| D | Cell cycle control, cell division, chromosome partitioning | 34 |
| E | Amino acid transport and metabolism | 124 |
| F | Nucleotide transport and metabolism | 86 |
| G | Carbohydrate transport and metabolism | 132 |
| H | Coenzyme transport and metabolism | 38 |
| I | Lipid transport and metabolism | 39 |
| J | Translation, ribosomal structure and biogenesis | 160 |
| K | Transcription | 134 |
| L | Replication, recombination and repair | 169 |
| M | Cell wall/membrane/envelope biogenesis | 107 |
| N | Cell motility | 9 |
| O | Posttranslational modification, protein turnover, chaperones | 37 |
| P | Inorganic ion transport and metabolism | 96 |
| Q | Secondary metabolites biosynthesis, transport and catabolism | 11 |
| R | General function prediction only | 0 |
| S | Function unknown | 316 |
| T | Signal transduction mechanisms | 33 |
| U | Intracellular trafficking, secretion, and vesicular transport | 46 |
| V | Defense mechanisms | 50 |
| W | Extracellular structures | 4 |
| Y | Nuclear structure | 0 |
| Z | Cytoskeleton | 0 |

**Table S2** **The phosphotransferase system (PTS) in the genome of *L. taiwanensis* CLG01.**

| **PTS transport system-raleted protein** | **Locus tag** |
| --- | --- |
| PTS glucose transporter subunit IIA | H1A07_02895,H1A07_04280 |
| PTS beta-glucoside transporter subunit IIBCA | H1A07_08640 |
| PTS galactitol transporter subunit IIC | H1A07_08970 |
| PTS cellobiose transporter subunit IIA | H1A07_01005 |
| PTS cellobiose transporter subunit IIC | H1A07_01010 |
| PTS lactose/cellobiose transporter subunit IIA | H1A07_01035 |
| PTS mannose/fructose/sorbose transporter family subunit IID | H1A07_08115,H1A07_08715 |
| PTS mannose/fructose/sorbose transporter subunit IIC | H1A07_08720 |
| PTS sugar transporter subunit IIC | H1A07_01050,H1A07_08120,H1A07_08835 |
| PTS sugar transporter subunit IIA | H1A07_00625,H1A07_00690,H1A07_08385 |
| PTS sugar transporter subunit IIB | H1A07_01045,H1A07_08125 |
| PTS glucitol/sorbitol transporter subunit IIA | H1A07_06770 |

**Table S3** **Detailed information of the bacteriocin biosynthetic gene cluster in *L. taiwanensis* CLG01 genome.**

| **Genes** | **From** | **to** | **Strands** | **Annotation** |
| --- | --- | --- | --- | --- |
| H1A07_02905 | 616,480 | 617,014 | + | PadR family transcriptional regulator |
| H1A07_02910 | 617,114 | 617,909 | + | DUF4097 family beta strand repeat protein |
| H1A07_02915 | 617,916 | 618,144 | + | hypothetical protein |
| H1A07_02920 | 618,170 | 618,806 | - | uridine kinase |
| H1A07_02925 | 619,252 | 620,578 | + | GHKL domain-containing protein |
| H1A07_02930 | 620,580 | 621,378 | + | response regulator transcription factor |
| H1A07_02935 | 621,390 | 623,550 | + | peptide cleavage/export ABC transporter |
| H1A07_02940 | 623,560 | 624,154 | + | HlyD family efflux transporter periplasmic adaptor subunit |
| H1A07_02945 | 624,280 | 624,508 | + | bacteriocin class II family protein |
| H1A07_02950 | 624,518 | 624,707 | + | bacteriocin |
| H1A07_02955 | 624,793 | 625,168 | + | hypothetical protein |
| H1A07_02960 | 625,527 | 626,577 | + | IS30 family transposase |
| H1A07_02965 | 626,830 | 626,911 | + | lactacin F inducer peptide precursor |
| H1A07_02970 | 627,098 | 628,298 | + | GHKL domain-containing protein |
| H1A07_02975 | 628,305 | 629,103 | + | response regulator transcription factor |

**Table S4 Detailed information of the LAP biosynthetic gene cluster in *L. taiwanensis* CLG01 genome.**

| **Genes** | **From** | **To** | **Strands** | **Annotation** |
| --- | --- | --- | --- | --- |
| H1A07_07335 | 1513425 | 1515669 | - | DNA helicase PcrA |
| H1A07_07340 | 1515768 | 1516395 | - | glycoside hydrolase family 73 protein |
| H1A07_07345 | 1516396 | 1517014 | - | ECF transporter S component |
| H1A07_07355 | 1517229 | 1517688 | - | SprT family protein |
| H1A07_07360 | 1517684 | 1518386 | - | glycosyl transferase |
| H1A07_07365 | 1518382 | 1519819 | - | oligosaccharide flippase family protein |
| H1A07_07370 | 1519830 | 1520979 | - | CDP-glycerol glycerophosphotransferase family protein |
| H1A07_07375 | 1521131 | 1521896 | - | ABC transporter permease |
| H1A07_07380 | 1521892 | 1522825 | - | ABC transporter ATP-binding protein |
| H1A07_07385 | 1523356 | 1524679 | - | YcaO-like family protein |
| H1A07_07390 | 1524668 | 1525613 | - | hypothetical protein |
| H1A07_07395 | 1525566 | 1526409 | - | SagB family peptide dehydrogenase |
| H1A07_07400 | 1526748 | 1527564 | - | helix-turn-helix transcriptional regulator |
| H1A07_07405 | 1527681 | 1530357 | - | calcium-translocating P-type ATPase, PMCA-type |
| H1A07_07410 | 1530528 | 1531362 | - | ammonia-dependent NAD(+) synthetase |
| H1A07_07415 | 1531363 | 1532836 | - | nicotinate phosphoribosyltransferase |
| H1A07_07420 | 1532951 | 1534946 | - | glycosyltransferase |
| H1A07_07425 | 1534929 | 1536045 | - | glycosyltransferase |

**Table S5 Detailed information of the Lanthipeptide biosynthetic gene cluster in *L. taiwanensis* CLG01 genome.**

| **Gene** | **From** | **To** | **Strands** | **Annotation** |
| --- | --- | --- | --- | --- |
| H1A07_08590 | 1742845 | 1743622 | - | DUF4931 domain-containing protein |
| H1A07_08595 | 1743772 | 1744195 | - | type I 3-dehydroquinate dehydratase |
| H1A07_08600 | 1744254 | 1745127 | - | cytochrome C5 |
| H1A07_08605 | 1745300 | 1746515 | + | MFS transporter |
| H1A07_08610 | 1746534 | 1747218 | + | type 1 glutamine amidotransferase |
| H1A07_08615 | 1747257 | 1748865 | - | ABC transporter ATP-binding protein |
| H1A07_08620 | 1748880 | 1749390 | - | O-acetyl-ADP-ribose deacetylase |
| H1A07_08625 | 1749849 | 1750644 | - | helix-turn-helix domain-containing protein |
| H1A07_08630 | 1750787 | 1752392 | - | ABC transporter ATP-binding protein |
| H1A07_08635 | 1752388 | 1754959 | - | hypothetical protein |
| H1A07_08640 | 1755351 | 1757295 | - | PTS beta-glucoside transporter subunit IIBCA |
| H1A07_08645 | 1757531 | 1759001 | + | sucrose-6-phosphate hydrolase |
| H1A07_08650 | 1759011 | 1759998 | + | LacI family DNA-binding transcriptional regulator |
| H1A07_08655 | 1760035 | 1760812 | - | tyrosine-protein phosphatase |
| H1A07_08660 | 1760995 | 1761811 | + | bifunctional hydroxymethylpyrimidine kinase/  phosphomethylpyrimidine kinase |
| H1A07_08665 | 1761886 | 1762570 | + | cobalamin biosynthesis protein CobQ |
| H1A07_08670 | 1762550 | 1763939 | + | amino acid permease |
| H1A07_08675 | 1763976 | 1764879 | - | EamA family transporter |


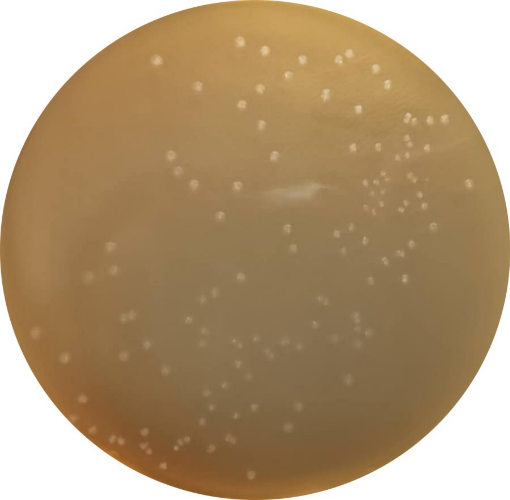


**Figure S1 Colony morphology of *L. taiwanensis* CLG01 on MRS agar plate after 48 h of incubation.**

**Figure S2 Morphology of *L. taiwanensis* CLG01 under a scanning electron microscope (SEM).**
